# Supplementary material for: Non-invasive tape sampling of tryptophan and kynurenine in relation to phenylalanine and tyrosine from melanoma and adjacent non-lesional skin: A pilot study
Source: PLoS One. 2025 Jun 24;20(6):e0326457. doi: 10.1371/journal.pone.0326457 (PMC12186910; doi:10.1371/journal.pone.0326457)
Supplement: S1 Fig — (DOCX) [file pone.0326457.s001.docx]

**S1 Fig. Pictures of tape sampling.** Adhesive sampling frame applied on melanoma lesion and adjacent NL skin to control for tape sampling area (a); D-squame tapes used for sampling (b); example of the 02F non-lesional skin and melanoma tape samples (c); examples of 06M patient 1^st^ tape strip samples under the light microscope after metabolites extraction (d).
