# Supplementary material for: Non-invasive tape sampling of tryptophan and kynurenine in relation to phenylalanine and tyrosine from melanoma and adjacent non-lesional skin: A pilot study
Source: PLoS One. 2025 Jun 24;20(6):e0326457. doi: 10.1371/journal.pone.0326457 (PMC12186910; doi:10.1371/journal.pone.0326457)
Supplement: S1 Table — (DOCX) [file pone.0326457.s002.docx]

**S1 Table**. **Parameters for Tyr, Phe, Trp and Kyn quantification by LC-MS/MS in dynamic multiple reaction monitoring (MRM) mode.** Retention time (RT), molecular weight (MW), selected precursor and product ion, and collision energies (CE).

| Analyte | RT | MW (Da) | Precursor ion | Precursor (m/z) | Product (m/z) | CE (eV) |  |
| --- | --- | --- | --- | --- | --- | --- | --- |
|  |  |  |  |  |  |  |  |
| L-tyrosine | 6.8 | 181.19 | [M+H] | 182.1 | 165.3 | 9 |  |
| L-tyrosine | 6.8 | 181.19 | [M+H] | 182.1 | 136.1 | 9 |  |
| L-tyrosine | 6.8 | 181.19 | [M+H] | 182.1 | 91.1 | 33 |  |
| [^2^H_4_]-L-tyrosine | 6.8 | 185.21 | [M+H] | 186.1 | 169.3 | 9 |  |
| [^2^H_4_]-L-tyrosine | 6.8 | 185.21 | [M+H] | 186.1 | 140.0 | 9 |  |
| L-phenylalanine | 10.2 | 165.19 | [M+H] | 166.1 | 120.1 | 13 |  |
| L-phenylalanine | 10.2 | 165.19 | [M+H] | 166.1 | 103.0 | 29 |  |
| L-phenylalanine | 10.2 | 165.19 | [M+H] | 166.1 | 76.9 | 45 |  |
| [^2^H_2_]-L-phenylalanine | 10.2 | 167.20 | [M+H] | 168.1 | 122.3 | 13 |  |
| [^2^H_2_]-L-phenylalanine | 10.2 | 167.20 | [M+H] | 168.1 | 104.3 | 29 |  |
| L-tryptophan | 10.6 | 204.23 | [M+H] | 205.1 | 188.1 | 9 |  |
| L-tryptophan | 10.6 | 204.23 | [M+H] | 205.1 | 118.3 | 49 |  |
| L-tryptophan | 10.6 | 204.23 | [M+H] | 205.1 | 91.2 | 40 |  |
| [^13^C_11_, ^12^N_2_]-L-tryptophan | 10.6 | 217.13 | [M+H] | 218.1 | 126.3 | 49 |  |
| [^13^C_11_, ^12^N_2_]-L-tryptophan | 10.6 | 217.13 | [M+H] | 218.1 | 98.2 | 40 |  |
| L-kynurenine | 9.8 | 208.22 | [M+H] | 209.1 | 192.1 | 5 |  |
| L-kynurenine | 9.8 | 208.22 | [M+H] | 209.1 | 146.0 | 21 |  |
| L-kynurenine | 9.8 | 208.22 | [M+H] | 209.1 | 94.0 | 9 |  |
| [^13^C_6_]-L-kynurenine | 9.8 | 214.17 | [M+H] | 215.1 | 198.3 | 5 |  |
| [^13^C_6_]-L-kynurenine | 9.8 | 214.17 | [M+H] | 215.11 | 152.3 | 21 |  |
