# Supplementary material for: Non-invasive tape sampling of tryptophan and kynurenine in relation to phenylalanine and tyrosine from melanoma and adjacent non-lesional skin: A pilot study
Source: PLoS One. 2025 Jun 24;20(6):e0326457. doi: 10.1371/journal.pone.0326457 (PMC12186910; doi:10.1371/journal.pone.0326457)
Supplement: S2 Table — (DOCX) [file pone.0326457.s003.docx]

**S2 Table. Evaluation of analytical LC-MS/MS method performance.** Repeatability of analytical method (a), and limit of detection (LOD) and quantification (LOQ).

(a) Repeatability of the analytical method used for Tyr, Phe, Trp and Kyn quantification. Calibration solutions were prepared for Tyr, Phe, Trp and Kyn with addition of the corresponding isotopically labelled internal standard (IS) at 0.25 µM for Phe, Trp, Kyn, and 0.75 µM for Tyr. The ratio of the peak areas (i.e., [Area]_is_/[Area]_Analyte_) was determined from three measurements performed within 48 hours. The repeatability is defined as coefficient of variation (CV (%)) of calibration standard measurements, *i.e.*, CV=SD/mean x 100%.

| Concentration (µM) | | CV (%) | | | |
| --- | --- | --- | --- | --- | --- |
| Phe, Trp, Kyn | Tyr | Phe | Trp | Kyn | Tyr |
| 0.0078 | 0.0234 | 3.2 | 3.5 | 0.7 | 1.8 |
| 0.063 | 0.188 | 2.5 | 0.4 | 0.8 | 0.3 |
| 0.125 | 0.375 | 3.1 | 2.4 | 2.0 | 0.7 |
| 0.50 | 1.50 | 3.1 | 4.8 | 2.4 | 0.5 |
| 2.00 | 6.00 | 0.6 | 1.6 | 2.0 | 0.2 |
| 4.00 | 12.0 | 1.6 | 3.5 | 4.5 | 0.9 |
| 8.00 | 24.0 | 2.2 | 0.9 | 1.2 | 1.8 |

(b) Limit of detection (LOD) and quantification (LOQ) of the analytical method. LOD and LOQ were determined based on linear regression analysis of the mean values of the calibration standards presented in (a) (n = 3; injection volume 15 µL). The LOD and LOQ were determined as follows: LOD = 3.3σ/slope and LOQ as LOQ = 10σ/slope, where σ is the standard error of the y-intercept from the regression analysis.

| Parameter | Concentration (µM) | | | |
| --- | --- | --- | --- | --- |
|  | Phe | Trp | Kyn | Tyr |
| LOD | 0.06 | 0.10 | 0.11 | 0.11 |
| LOQ | 0.18 | 0.31 | 0.35 | 0.33 |
