# Supplementary material for: Non-invasive tape sampling of tryptophan and kynurenine in relation to phenylalanine and tyrosine from melanoma and adjacent non-lesional skin: A pilot study
Source: PLoS One. 2025 Jun 24;20(6):e0326457. doi: 10.1371/journal.pone.0326457 (PMC12186910; doi:10.1371/journal.pone.0326457)
Supplement: S1 Section — (DOCX) [file pone.0326457.s004.docx]

**S1 Section. Preparation of Tyr, Phe, Trp and Kyn stock solutions.**

Stock solutions of Trp, 4.08 mg/mL (20mM), Kyn, 4.16 mg/mL (20mM), and Phe, 1mg/mL (6.05 mM) were prepared in MilliQ water. Stock solution of Tyr was prepared by dissolving it in 99.9% formic acid, followed by dilution with MilliQ water to reach 1mg/mL (5.52 mM) concentration (final concentration of formic acid did not exceed 1% v/v). Stock solutions of isotopically labelled Trp, 2.043mg/mL (9.46 mM), and Kyn, 2.08 mg/mL (9.36 mM) were prepared in 50% acetonitrile in MilliQ water (v/v). Stock solution of isotopically labelled Tyr, 1.85 mg/mL (12.17 mM) was prepared in 1M HCl, and Phe, 1mg/mL (5.98 mM) – in 10% methanol in MilliQ water (v/v). Dilution series for calibration curves were prepared in solution consisting of 10% methanol in water.
