# Supplementary material for: Non-invasive tape sampling of tryptophan and kynurenine in relation to phenylalanine and tyrosine from melanoma and adjacent non-lesional skin: A pilot study
Source: PLoS One. 2025 Jun 24;20(6):e0326457. doi: 10.1371/journal.pone.0326457 (PMC12186910; doi:10.1371/journal.pone.0326457)
Supplement: S2 Fig — (DOCX) [file pone.0326457.s005.docx]

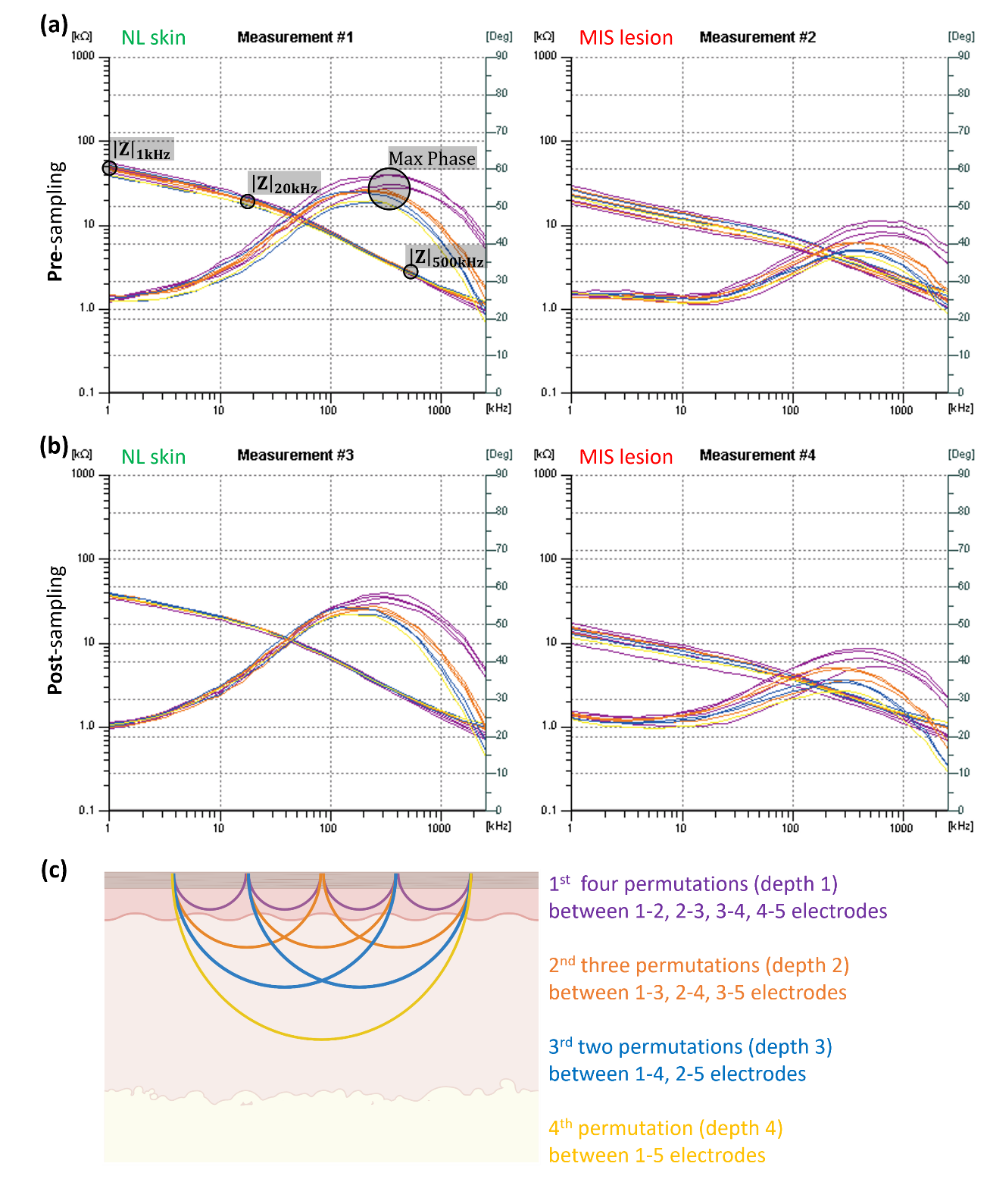


**S2 Fig. Representative impedance data obtained by Nevisense measurement.** NL skin and MIS lesion before (a) and after (b) tape sampling (04F patient). The measurements are displayed as curves of magnitude (kΩ, left y axis) and phase shift (degrees, right y axis) at four different depths (different colors) and 10 permutations at various frequences (x axis). Schematic skin illustration showing 10 permutations measured between the Nevisense electrodes, generating four different measurement depths. Illustration adapted from Nevisense clinical reference guide[1] and created with BioRender.com (c). The absolute impedance obtained at the lowest 1 kHz frequency, i.e., IZI_1kHz_ reflects resistive properties of the main skin barrier stratum corneum (SC) layer associated with ion-conductive pathways in the SC, e.g., extracellular and intracellular routes and appendages, such as sweat ducts and hair follicles[2]. Impedance magnitudes at frequencies of 20 and 500 kHz were used to determine magnitude index (MIX), which can be defined as MIX=IZI_20kHz_/IZI_500kHz_[3]. MIX reflects both resistive properties of the skin and capacitive properties of skin associated with restriction of ion transfer at low conductive lipid and lipid-protein domains in SC (e.g., extracellular lipid lamellae and keratin properties)[2]. Max Phase corresponds to the maximum value of the phase peak (in degrees) and it is related to the capacitive properties of the SC. IZI at 1kHz, MIX and Max Phase values are averaged values over all permutations/depths.

**Reference**

1. Nevisense Clinical Reference Guide. The EIS Method and How to Interpret the Nevisense Result. 2014. doi:Article Number: 975-0009-04

2. Björklund S, Ruzgas T, Nowacka A, Dahi I, Topgaard D, Sparr E, et al. Skin membrane electrical impedance properties under the influence of a varying water gradient. Biophys J. 2013;104: 2639–2650. doi:10.1016/j.bpj.2013.05.008

3. Morin M, Ruzgas T, Svedenhag P, Anderson CD, Ollmar S, Engblom J, et al. Skin hydration dynamics investigated by electrical impedance techniques in vivo and in vitro. Sci Rep. 2020;10: 17218. doi:10.1038/s41598-020-73684-y
