# Supplementary material for: Non-invasive tape sampling of tryptophan and kynurenine in relation to phenylalanine and tyrosine from melanoma and adjacent non-lesional skin: A pilot study
Source: PLoS One. 2025 Jun 24;20(6):e0326457. doi: 10.1371/journal.pone.0326457 (PMC12186910; doi:10.1371/journal.pone.0326457)
Supplement: S3 Table — (DOCX) [file pone.0326457.s006.docx]

**S3 Table**. **Normality assessment of paired differences using Shapiro-Wilk test.** Tests were performed on raw data (RD) and data after outliers removal (OR).

1. Amounts of tyrosine (Tyr), phenylalanine (Phe), tryptophan (Trp), kynurenine and their ratios (Fig 4 A-B).

| Analyte | Shapiro Wilk normality test (RD/OR) | | |
| --- | --- | --- | --- |
|  | NL-MM | NL-MIS | NL-BL |
| Tyr | p=0.015/0.534 | p=0.567/0.682 | p= 0.995/0.995 |
| Phe | p=0.222/0.388 | p= 0.360/0.219 | p=0.584/0.584 |
| Trp | p=0.779/0.779 | p=0.74640.434 | p=0.786/0.786 |
| Kyn | p=0.224/0.455 | p=0.282/0.282 | p=0.119/0.119 |
| Trp/Tyr | p=0.977/0.977 | p=0.305/0.459 | p=0.208/0.208 |
| Trp/Phe | p=0.671/0.671 | p=0.288/0.288 | p=0.304/0.304 |
| Phe/Tyr | p=0.532/0.532 | p=0.488/0.488 | p=0.906/0.906 |
| Trp/Kyn | p=0.029/0.359 | p=0.202/0.286 | p=0.717/0.717 |
| Trpnorm/Kynnorm | p=0.131/0.594 | p=0.525/0.704 | p=0.006/0.1186 |

1. Skin resistance, IZI at 1 kHz (Fig 6A).

| Sample | Shapiro Wilk normality test (RD/OR) | | |
| --- | --- | --- | --- |
|  | NL-MM | NL-MIS | NL-BL |
| Pre | p=0.477/0.477 | p=0.815/0.815 | p= 0.941/0.941 |
| Post | p=0.211/0.214 | p= 0.274/0.855 | p=0.725/0.725 |

1. Skin resistance at 1 kHz (Fig 6B).

| Sample | Shapiro Wilk normality test (RD/OR) | | | |
| --- | --- | --- | --- | --- |
|  | MM | MIS | BL | NL |
| Pre vs post | p=0.994/0.988 | p=0.882/0.882 | p=0.299/0.299 | p=0.526/0.299 |
