# Supplementary material for: Non-invasive tape sampling of tryptophan and kynurenine in relation to phenylalanine and tyrosine from melanoma and adjacent non-lesional skin: A pilot study
Source: PLoS One. 2025 Jun 24;20(6):e0326457. doi: 10.1371/journal.pone.0326457 (PMC12186910; doi:10.1371/journal.pone.0326457)
Supplement: S2 Section — (DOCX) [file pone.0326457.s007.docx]

**S2 Section**. **Diagnostic melanoma classification**.

Cutaneous melanoma arises from uncontrolled proliferation of melanocytes present in the basal layer of the epidermis[1]. Melanoma is a neoplasm that evolves through several stages distinguished by clinical and pathological features[2]. Melanocytic nevi, commonly known as moles, are benign proliferation of melanocytes, with a very low likelihood of progressing to melanoma[3]. Dysplastic nevi is enlarged nevi with different severity of dysplasia[3]. Dysplastic nevi, in most cases is considered as benign lesion (BL), however dysplastic nevi sometimes is also seen as intermediate category in the progression between benign and malignant lesion. Due to the lack of information regarding the dysplastic nevi transition to malignant lesions, currently clinicians are inconsistent with dysplastic nevi diagnosis, grading and treatment[3]. Melanoma in situ (MIS) refers to proliferation of atypical melanocytes within the epidermis. Melanoma in situ is a malignant tumor which is confined to the epidermis without dermal invasion. Invasive melanoma, in this study referred to as malignant melanoma (MM), occurs when cancer growth penetrates deeper dermal layers of the skin and spreads to other parts of the body where metastatic cancer may occur as a progression of the invasive cancer[3]. Melanoma patients can be classified into five distinct stages where stage 0 is defined as melanoma in situ, stages I and II identify localized disease, while III and IV diagnose disease progression with nodal or distant metastases, respectively[1]. Primary tumors are also divided into four classes (pT1-pT4) based on tumor thickness, and subdivided in a or b which stands for absence or presence of ulcerations[4,5]. The most common melanoma subtypes are superficial spreading melanoma (SMM), nodular melanoma (NM) and lentigo maligna melanoma (LMM)[6]. SMM most common melanoma subtype (70%), presenting flat, slowly growing lesion. NM accounts for 20% of all melanomas, and it grows as nodule, which may be pigmented or amelanotic. LMM represent 5-10% of melanomas and it manifest as flat macules on the face of older patients[6]. The survival analysis performed on clinical data from several countries[6,7], including Sweden[8], indicates that NM melanoma subtype has worse survival rate compared to SMM, whereas prognostic values of LMM is not very clear[6–8]. The overall formation of melanoma diagnosis is dependent on the clinical factors such as tumor thickness, ulceration, sun damage, growth phase etc., and is very important for evaluation of level of aggressiveness[9].

**References**

1. Ward WH, Jeffrey M. Farma. Cutaneous melanoma Etiology and Therapy. doi:http://dx.doi.org/10.15586/codon.cutaneousmelanoma.2017

2. Taylor NJ, Gaynanova I, Eschrich SA, Welsh EA, Garrett TJ, Beecher C, et al. Metabolomics of primary cutaneous melanoma and matched adjacent extratumoral microenvironment. PLoS One. 2020;15: 1–24. doi:10.1371/journal.pone.0240849

3. Shain AH, Bastian BC. From melanocytes to melanomas. Nat Rev Cancer. 2016;16: 345–58. doi:10.1038/nrc.2016.37

4. Karagiannis P, Fittall M, Karagiannis SN. Evaluating biomarkers in melanoma. Front Oncol. 2015;4: 1–11. doi:10.3389/fonc.2014.00383

5. Puglisi R, Bellenghi M, Pontecorvi G, Pallante G, Carè A, Mattia G. Biomarkers for diagnosis, prognosis and response toimmunotherapy in melanoma. Cancers (Basel). 2021;13. doi:10.3390/cancers13122875

6. Sharouni M El, Diest PJ Van, Witkamp AJ, Gils CH Van. Subtyping Cutaneous Melanoma Matters. 2020;4: 1–6. doi:10.1093/jncics/pkaa097

7. Lattanzi M, Lee Y, Simpson D, Moran U, Darvishian F, Randie H, et al. Primary Melanoma Histologic Subtype : Impact on Survival and Response to Therapy. 2019;111: 5–12. doi:10.1093/jnci/djy086

8. Andersson R, Dufmats M, Sc B, Hansson J, Ph D, Ingvar C, et al. Invasive Cutaneous Malignant Melanoma in Sweden , A Prospective , Population-Based Study of Survival and Prognostic Factors. 2004; 1990–1999. doi:10.1002/cncr.20602

9. Chatzilakou E, Hu Y, Jiang N, Yetisen AK. Biosensors and Bioelectronics Biosensors for melanoma skin cancer diagnostics. Biosens Bioelectron. 2024;250: 116045. doi:10.1016/j.bios.2024.116045
