# Supplementary material for: Non-invasive tape sampling of tryptophan and kynurenine in relation to phenylalanine and tyrosine from melanoma and adjacent non-lesional skin: A pilot study
Source: PLoS One. 2025 Jun 24;20(6):e0326457. doi: 10.1371/journal.pone.0326457 (PMC12186910; doi:10.1371/journal.pone.0326457)
Supplement: S4 Table — (DOCX) [file pone.0326457.s008.docx]

**S4 Table**. **Statistical evaluation of age, sampling size and site distribution between the study participants.**

(a) Mean values and standard deviation (mean±SD) of age and sampling size within the groups of the study participants.

| Groups | Age (years) | Size (cm^2^) |
| --- | --- | --- |
| BL (n=3) | 62.0±18.3 | 0.5±0.2 |
| MIS (n=6) | 73.0±14.8 | 1.0±0.6 |
| MM (n=7) | 62.9±15.6 | 1.4±0.8 |
| Female (n=6) | 67.3±21.5 | 1.1±0.5 |
| Male (n=10) | 66.0±12.1 | 1.1±0.8 |

(b) Contingency tables of sampling sites distribution between females and males, and BL, MIS and MM study participants.

| Group | Abdominal | Arm | Back | Chest | Groin | Thigh |
| --- | --- | --- | --- | --- | --- | --- |
| Female | 1 | 1 | 3 | 0 | 0 | 1 |
| Male | 0 | 2 | 4 | 2 | 1 | 1 |
| BL | 0 | 0 | 2 | 0 | 1 | 0 |
| MIS | 1 | 2 | 2 | 1 | 0 | 0 |
| MM | 0 | 1 | 3 | 1 | 0 | 2 |

(c) Comparison between females and males, and BL, MIS and MM, in terms of age, sampling size and site. The p-values for age and size were obtained from a one-way ANOVA with Tukey's post hoc test, and for sites from Fisher’s exact test.

| Groups | Age | Size | Site |
| --- | --- | --- | --- |
| MM-MIS | p=0.495 | p=0.591 | p=0.790 |
| BL-MIS | p=0.595 | p=0.582 | p=0.571 |
| BL-MM | p=0.997 | p=0.195 | p=0.833 |
| Female-male | p=0.875 | p=0.915 | p=0.853 |
