# Supplementary material for: Non-invasive tape sampling of tryptophan and kynurenine in relation to phenylalanine and tyrosine from melanoma and adjacent non-lesional skin: A pilot study
Source: PLoS One. 2025 Jun 24;20(6):e0326457. doi: 10.1371/journal.pone.0326457 (PMC12186910; doi:10.1371/journal.pone.0326457)
Supplement: S5 Table — (DOCX) [file pone.0326457.s009.docx]

**S5 Table. Average amounts of analytes and their determined ratios from study participants.** Malignant melanoma (MM), melanoma in situ (MIS), benign lesions (BL) and adjacent non-lesional skin (NL) patient skin samples. Mean values and standard deviation (mean±SD) were calculated from the raw data (RD) and after removal of statistical outliers (OR).

(a) Amounts

| Sample | Amount (nmol/cm^2^) | | | |
| --- | --- | --- | --- | --- |
|  | Tyr | Phe | Trp | Kyn x10^-3^ |
| NL_RD_/NL_OR_ | 2.2±1.2  (n=7 / 7) | 0.8±0.4  (n=7 / 7) | 0.6±0.4  (n=7 / 7) | 2.1±1.7 /1.5±0.8  (n=7 / 6) |
| MM_RD_/MM_OR_ | 7.3±2.2 /6.6±1.2 (n=7 / 6) | 3.1±0.9 / 3.1±0.3 (n=7 / 5) | 2.2±0.6  (n=7 / 7) | 3.5±1.6  (n=7 / 7) |
| NL_RD_/NL_OR_ | 4.4±2.4  (n=6 / 6) | 1.5±0.8  (n=6 / 6) | 1.3±0.8  (n=6 / 6) | 3.1±1.4  (n=6 / 6) |
| MIS_RD_/MIS_OR_ | 6.3±5.6 / 4.2±2.5 (n=6 / 5) | 2.3±1.4 / 1.8±0.6 (n=6 / 5) | 1.6±1.4 / 1.0±0.4 (n=6 / 5) | 3.3±2.02  (n=6 / 6) |
| NL_RD_/NL_OR_ | 2.2±1.7  (n=3 / 3) | 0.7±0.4  (n=3 / 3) | 0.8±0.6  (n=3 / 3) | 2.4±1.2  (n=3 / 3) |
| BL_RD_/BL_OR_ | 2.2±2.4  (n=3 /3) | 0.8± 0.7  (n=3 / 3) | 0.7±0.7  (n=3 / 3) | 1.9±0.7  (n=3 / 3) |

(b) Ratios

| Sample | Ratio | | | |  |
| --- | --- | --- | --- | --- | --- |
|  | Trp/Tyr | Trp/Phe | Phe/Tyr | Trp/Kyn x10^3^ | Trp_norm_/Kyn_norm_ |
| NL_RD_/NL_OR_ | 0.29±0.04  (n=7 / 7) | 0.8±0.1  (n=7 / 7) | 0.4±0.1  (n=7 / 7) | 0.4±0.2  (n=7 / 7) | 1.9±1.8 / 1.2±0.6  (n=7 / 6) |
| MM_RD_/MM_OR_ | 0.30±0.04  (n=7 / 7) | 0.7±0.1  (n=7 / 7) | 0.4±0.1  (n=7 / 7) | 0.7±0.2 /0.7±0.1  (n=7 / 6) | 0.7±0.3  (n=7 / 7) |
| NL_RD_/NL_OR_ | 0.30±0.02  (n=6 / 6) | 0.9±0.2  (n=6 / 6) | 0.4±0.1  (n=6 / 6) | 0.4±0.2 / 0.4±0.1  (n=6 / 5) | 1.1±0.7 / 0.8±0.3  (n=6 / 5) |
| MIS_RD_/MIS_OR_ | 0.27±0.05 / 0.29±0.02 (n=6 / 5) | 0.6±0.2  (n=6 / 6) | 0.4±0.1  (n=6 / 6) | 0.5±0.2  (n=6 / 6) | 0.7±0.4 / 0.6±0.2  (n=6 / 5) |
| NL_RD_/NL_OR_ | 0.39±0.05  (n=3 / 3) | 1.0±0.2  (n=3 / 3) | 0.4±0.1  (n=3 / 3) | 0.3±0.2  (n=3 / 3) | 1.3±0.5  (n=3 / 3) |
| BL_RD_/BL_OR_ | 0.36±0.04  (n=3 / 3) | 0.8± 0.1  (n=3 / 3) | 0.5±0.1  (n=3 / 3) | 0.3±0.3  (n=3 / 3) | 1.3±0.65  (n=3 / 3) |
