# Supplementary material for: Non-invasive tape sampling of tryptophan and kynurenine in relation to phenylalanine and tyrosine from melanoma and adjacent non-lesional skin: A pilot study
Source: PLoS One. 2025 Jun 24;20(6):e0326457. doi: 10.1371/journal.pone.0326457 (PMC12186910; doi:10.1371/journal.pone.0326457)
Supplement: S6 Table — (DOCX) [file pone.0326457.s010.docx]

**S6 Table**. **Compilation of p-values from statistical analyses.** Analyses performed on analyte abundance (a) and their ratios (b) collected from benign lesions (BL), melanoma in situ (MIS), malignant melanoma (MM), and adjacent non-lesional (NL) skin, as presented in Fig 4 of the main article. Non-adjusted p-value obtained from paired sample t-test or from non-parametric Wilcoxon matched pairs test in cases where assumption of normal distribution was not fulfilled, S3 Table. P-values were corrected for multiple testing by false discovery rate (p_fdr_) corrections. False discovery rate correction (also known as Benjamini-Hochberg correction) was performed by using ‘fdr’ function implemented in basic R program. The significance levels used were: *p < 0.05,**p < 0.01. Statistical analysis performed on raw data (RD) and after outliers removal (OR).

1. Amounts

| Sample | RD/OR | | | |
| --- | --- | --- | --- | --- |
|  | Tyr | Phe | Trp | Kyn |
| NL-MM | p=0.003/0.003 | p=0.001/0.001 | p=0.001/0.001 | p=0.178/0.008 |
|  | p_fdr_=0.009/0.008 | p_fdr_=0.006/0.006 | p_fdr_=0.006/0.006 | p_fdr_=0.200/0.015 |
| NL-MIS | p=0.381/0.844 | p=0.191/0.379 | p=0.636/0.645 | p=0.797/0.797 |
|  | p_fdr_=0.572/0.844 | p_fdr_=0.375/0.696 | p_fdr_=0.715/0.830 | p_fdr_=0.797/0.844 |
| NL-BL | p=0.979 | p=0.689 | p=0.791 | p=0.311 |
|  | p_fdr_=0.996 | p_fdr_=0.996 | p_fdr_=0.996 | p_fdr_=0.700 |

(b) Ratios

| Sample | RD/OR | | | | |  |
| --- | --- | --- | --- | --- | --- | --- |
|  | Trp/Tyr | Trp/Phe | Phe/Tyr | Trp/Kyn | Trp_norm_/Kyn_norm_ | |
| NL-MM | p=0.674/0.674 | p=0.008/0.008 | p=0.119/0.119 | p=0.016/0.019 | p=0.094/0.108 |  |
|  | p_fdr_=0.674/0.674 | p_fdr_=0.018/0.015 | p_fdr_=0.153/0.134 | p_fdr_=0.028/0.029 | p_fdr_=0.144/0.134 |  |
| NL-MIS | p=0.208/0.387 | p=0.052/0.052 | p=0.115/0.115 | p=0.554/0.615 | p=0.179/0.368 |  |
|  | p_fdr_=0.375/0.696 | p_fdr_=0.375/0.465 | p_fdr_=0.375/0.518 | p_fdr_=0.713/0.830 | p_fdr_=0.375/0.696 |  |
| NL-BL | p=0.064 | p=0.092 | p=0.231 | p=0.587 | p=0.996 |  |
|  | p_fdr_=0.414 | p_fdr_=0.414 | p_fdr_=0.694 | p_fdr_=0.996 | p_fdr_=0.996 |  |
