# Supplementary material for: Non-invasive tape sampling of tryptophan and kynurenine in relation to phenylalanine and tyrosine from melanoma and adjacent non-lesional skin: A pilot study
Source: PLoS One. 2025 Jun 24;20(6):e0326457. doi: 10.1371/journal.pone.0326457 (PMC12186910; doi:10.1371/journal.pone.0326457)
Supplement: S7 Table — (DOCX) [file pone.0326457.s011.docx]

**S7 Table. Ratios between melanoma-suspected lesion and non-lesional skin**. Ratio 1 indicates no differences between the NL and lesional samples. Brackets [] indicates statistical outliers. Mean values and standard deviation were calculated by using raw data (RD) and after outliers’ removal (OR).

| Diagnosis | Patient | Tyr | Phe | Trp | Kyn | Trp/Tyr | Trp/Phe | Phe/Tyr | Trp/Kyn |
| --- | --- | --- | --- | --- | --- | --- | --- | --- | --- |
| MM | 02F | 2.8 | 3.3 | 3.2 | 3.0 | 1.1 | 1.0 | 1.2 | 1.1 |
|  | 03F | 3.0 | 3.8 | 3.2 | 2.0 | 1.1 | 0.8 | 1.3 | 1.6 |
|  | 03M | 6.1 | 7.3 | 5.8 | 4.1 | 1.0 | 0.8 | 1.2 | 1.4 |
|  | 04M | [19.8] | [16.0] | [15.8] | [6.5] | 0.8 | 1.0 | [0.8] | 2.4 |
|  | 06F | 3.4 | 4.2 | 3.6 | 1.9 | 1.0 | 0.9 | 1.2 | 1.9 |
|  | 08M | 2.1 | 3.2 | 2.8 | 0.4 | 1.4 | 0.9 | [1.5] | [8.0] |
|  | 10M | 1.7 | 1.8 | 1.6 | 1.5 | 0.9 | 0.9 | 1.0 | 1.0 |
|  | Mean±SD (RD) | 5.6±6.4 (n=7) | 5.6±4.9 (n=7) | 5.1±4.9 (n=7) | 2.7±2.0 (n=7) | 1.0±0.2 (n=7) | 0.9±0.1 (n=7) | 1.2±0.2 (n=7) | 2.5±2.5 (n=7) |
|  | Mean±SD (OR) | 3.2±1.6 [n=6] | 3.9±1.8 [n=6] | 3.4±1.4 [n=6] | 2.1±1.3 [n=6] | 1.0±0.2 (n=7) | 0.9±0.1 (n=7) | 1.2±0.1 [n=5] | 1.6±0.5 [n=6] |
| MIS | 01M | 0.6 | 1.0 | 0.5 | 0.6 | 0.9 | 0.5 | [1.7] | 0.8 |
|  | 04F | 2.5 | 2.3 | 2.2 | 2.1 | 0.9 | 0.9 | 0.9 | 1.0 |
|  | 05F | 2.5 | 2.7 | 2.7 | 1.5 | 1.1 | 1.0 | 1.1 | [1.8] |
|  | 05M | 0.7 | 0.9 | 0.7 | 0.7 | 1.0 | 0.8 | 1.3 | 1.0 |
|  | 09M | 0.6 | 0.8 | 0.6 | 0.5 | 1.0 | 0.8 | 1.3 | 1.2 |
|  | 06M | 2.4 | 2.4 | 1.4 | 2.0 | [0.6] | 0.6 | 1.0 | 0.7 |
|  | Mean±SD (RD) | 1.6±1.0 (n=6) | 1.7±0.9 (n=6) | 1.3±0.9 (n=6) | 1.2±0.7 (n=6) | 0.9±0.2 (n=6) | 0.8±0.2 (n=6) | 1.2±0.3 (n=6) | 1.1±0.4 (n=6) |
|  | Mean±SD (OR) | 1.6±1.0 (n=6) | 1.7±0.9 (n=6) | 1.3±0.9 (n=6) | 1.2±0.7 (n=6) | 1.0±0.1 [n=5] | 0.8±0.2 (n=6) | 1.1±0.2 [n=5] | 1.0±0.2 [n=5] |
| BL | 01F | 0.5 | 0.7 | 0.5 | 0.6 | 1.0 | 0.7 | 1.3 | 0.8 |
|  | 02M | 1.0 | 1.0 | 0.9 | 0.9 | 0.9 | 0.9 | 1.0 | 1.1 |
|  | 07M | 1.2 | 1.5 | 1.2 | 1.0 | 1.0 | 0.8 | 1.2 | 1.2 |
|  | Mean±SD (RD) | 0.9±0.4 (n=3) | 1.1±0.4 (n=3) | 0.9±0.3 (n=3) | 0.8±0.2 (n=3) | 0.9±0.02 (n=3) | 0.8±0.1 (n=3) | 1.2±0.2 (n=3) | 1.0±0.2 (n=3) |
|  | Mean±SD (OR) | 0.9±0.4 (n=3) | 1.1±0.4 (n=3) | 0.9±0.3 (n=3) | 0.8±0.2 (n=3) | 0.9±0.02 (n=3) | 0.8±0.1 (n=3) | 1.2±0.2 (n=3) | 1.0±0.2 (n=3) |
