# Supplementary material for: Non-invasive tape sampling of tryptophan and kynurenine in relation to phenylalanine and tyrosine from melanoma and adjacent non-lesional skin: A pilot study
Source: PLoS One. 2025 Jun 24;20(6):e0326457. doi: 10.1371/journal.pone.0326457 (PMC12186910; doi:10.1371/journal.pone.0326457)
Supplement: S8 Table — (DOCX) [file pone.0326457.s012.docx]

**S8 Table**. **Comparisons of skin resistance between the samples.** Pre- and post-sampling skin resistance was measured on benign lesions (BL, n=3), melanoma ins situ (MIS, n=6), malignant melanoma (MM, n=7) and adjacent non-lesional (NL, n=16) skin. Data is presented in Figure 4 in the main article. Compilation of the p-values. Non-adjusted p-value obtained from paired sample t-test corrected for multiple testing by false discovery rate (p_fdr_) corrections. False discovery rate correction (also known as Benjamini-Hochberg correction) was performed by using ‘fdr’ function implemented in basic R program. The significance levels used were: *p < 0.05,**p < 0.01. Statistical analysis performed on raw data (RD) and after outliers removal (OR).

(a) Comparison between melanoma lesions and their adjacent NL skin in terms of skin resistance. p-values obtained from the paired two-sample t-test.

| Skin sample | Mean±SD (RD/OR) | | p-values (p/pfdr) | | |
| --- | --- | --- | --- | --- | --- |
|  |  |  | Pre-sampling | Post-sampling | |
|  | Pre-sampling | Post-sampling | RD | RD | OR |
| NL | 80.3±30.4 | 53.1±24.8/56.6±25.3 | 0.006/0.017 | 0.229/0.344 | 0.122/0.366 |
| MM | 35.4±16.3 | 34.3±26.7/25.4±14.2 |  |  |  |
| NL | 86.4± 45.7 | 55.7±35.0/46.7±4.6 | 0.538/0.538 | 0.425/0.538 | 0.915/0.915 |
| MIS | 69.0±57.8 | 36.8±27.6/48.5±26.8 |  |  |  |
| NL | 75.0±64.3 | 65.7±19.1 | 0.356/0.535 | 0.010/0.031 |  |
| BL | 44.6±27.2 | 40.9±15.0 |  |  |  |

(b) Comparison between pre- and post-sampling. p-values obtained from the two-sample t-test

| Skin sample | \|Z\| at 1kHz (kOhm) | | |
| --- | --- | --- | --- |
|  | *Pre-sampling* | *Post-sampling* | *p*-values (p/p_fdr_) |
| NL (RD) | 81.6±40.4 | 56.5±27.0 | 0.006 |
| NL (OR) | 72.7±34.6 | 49.4±19.0 | 0.009 |
| BL (RD) | 44.6± 27.2 | 40.9±15.0 | 0.668/0.668 |
| BL (OR) | 44.6± 27.2 | 40.9±15.0 | 0.668/0.668 |
| MIS (RD) | 69.0± 57.8 | 36.8±27.6 | 0.093/0.279 |
| MIS (OR) | 69.0± 57.8 | 36.8±27.6 | 0.093/0.279 |
| MM (RD) | 35.1± 16.3 | 34.3± 26.7 | 0.933/0.933 |
| MM (OR) | 33.2±16.9 | 25.4±14.2 | 0.409/0.409 |
