# Supplementary material for: Non-invasive tape sampling of tryptophan and kynurenine in relation to phenylalanine and tyrosine from melanoma and adjacent non-lesional skin: A pilot study
Source: PLoS One. 2025 Jun 24;20(6):e0326457. doi: 10.1371/journal.pone.0326457 (PMC12186910; doi:10.1371/journal.pone.0326457)
Supplement: S4 Fig — (DOCX) [file pone.0326457.s014.docx]

|  |
| --- |
| **S4 Fig. Principal component analysis (PCA) (a) and Partial least squares-discriminant analysis (PLS-DA) (b) of melanoma patients.** Score plot (a1) and variable loadings (a2) for PCA analysis. Score plot of PLS-DA performed by using response variable (Y) of four groups (b, g), i.e., malignant melanoma (MM), melanoma in situ (MIS), benign lesions (BL), non-lesional skin (NL) and two groups (b1). Contribution of variables to PLS-DA analysis ranked by VIP scores and each variable contribution to the response outcome, i.e., NL, BL, MIS and MM, visualized by heat map (b2). Q^2^Y measure of the predictive performance of the model R^2^X and R^2^Y is measure of proportion of the variance explained by predictor variable (X) and response variable (Y). Q^2^Y measure of the predictive performance of the model. MIX stands for the average gradient slope of magnitude between 20kHz and 500kHz; MaxPhase-the maximum peak of the phase in degrees; IZI-impedance at 1 kHz/skin resistance. |
