# Supplementary material for: Non-invasive tape sampling of tryptophan and kynurenine in relation to phenylalanine and tyrosine from melanoma and adjacent non-lesional skin: A pilot study
Source: PLoS One. 2025 Jun 24;20(6):e0326457. doi: 10.1371/journal.pone.0326457 (PMC12186910; doi:10.1371/journal.pone.0326457)
Supplement: S5 Fig — (DOCX) [file pone.0326457.s015.docx]

|  |
| --- |
| **S5 Fig. Images showing sampling areas larger than melanoma lesions.** The percentage on the image indicates the percentage of NL skin area sampled together with MIS lesions. |
