# Supplementary material for: Non-invasive tape sampling of tryptophan and kynurenine in relation to phenylalanine and tyrosine from melanoma and adjacent non-lesional skin: A pilot study
Source: PLoS One. 2025 Jun 24;20(6):e0326457. doi: 10.1371/journal.pone.0326457 (PMC12186910; doi:10.1371/journal.pone.0326457)
Supplement: Attachment — Submitted filename: Response to Reviewers_20250503.pdf [file pone.0326457.s020.pdf]

Comments to the Author

1. If the authors have adequately addressed your comments raised in a previous round of review and you feel that this manuscript is now acceptable for publication, you may indicate that here to bypass the “Comments to the Author” section, enter your conflict of interest statement in the “Confidential to Editor” section, and submit your "Accept" recommendation.

Reviewer #1: All comments have been addressed

Reviewer #3: All comments have been addressed

____

2. Is the manuscript technically sound, and do the data support the conclusions?

The manuscript must describe a technically sound piece of scientific research with data that supports the conclusions. Experiments must have been conducted rigorously, with appropriate controls, replication, and sample sizes. The conclusions must be drawn appropriately based on the data presented.

Reviewer #1: Yes

Reviewer #3: Partly

____

3. Has the statistical analysis been performed appropriately and rigorously?

Reviewer #1: I Don't Know

Reviewer #3: Yes

____

4. Have the authors made all data underlying the findings in their manuscript fully available?

The PLOS Data policy requires authors to make all data underlying the findings described in their manuscript fully available without restriction, with rare exception (please refer to the Data Availability Statement in the manuscript PDF file). The data should be provided as part of the manuscript or its supporting information, or deposited to a public repository. For example, in addition to summary statistics, the data points behind means, medians and variance measures should be available. If there are restrictions on publicly sharing data—e.g. participant privacy or use of data from a third party—those must be specified.

Reviewer #1: Yes

Reviewer #3: Yes

____

5. Is the manuscript presented in an intelligible fashion and written in standard English?

PLOS ONE does not copyedit accepted manuscripts, so the language in submitted articles must be clear, correct, and unambiguous. Any typographical or grammatical errors should be corrected at revision, so please note any specific errors here.

Reviewer #1: Yes

Reviewer #3: Yes

---

6. Review Comments to the Author

Please use the space provided to explain your answers to the questions above. You may also include additional comments for the author, including concerns about dual publication, research ethics, or publication ethics. (Please upload your review as an attachment if it exceeds 20,000 characters)

Reviewer #1: The authors have answered all my questions in a satisfying way and I my opinion the paper is ready for publication.

Reviewer #3: The manuscript presents an interesting follow-up study investigating the metabolic alterations of tryptophan (Trp) and kynurenine (Kyn) in melanoma lesions using non-invasive tape sampling. The study builds upon the authors' previous work and explores whether a higher Kyn abundance and lower Trp abundance can be detected on the surface of cutaneous melanoma lesions compared with adjacent non-lesional skin. While the study is methodologically novel and clinically relevant, several concerns need to be addressed:

1. The authors should clearly define the inclusion and exclusion criteria in the Methods section. Individuals with melanoma or similar skin lesions often have other coexisting dermatological conditions due to shared risk factors such as UV exposure, genetic predisposition, and immunosuppression. These conditions, including actinic keratosis, seborrheic keratosis, dysplastic nevi, and inflammatory skin diseases such as psoriasis or atopic dermatitis, may confound the metabolic profiles observed in the study. The authors should clarify whether such conditions were excluded or controlled for in the study design.

Thank you, we agree with the reviewer. However, since this was a pilot study with limited number of study participants, no other exclusion criteria than lesion size was applied. Clarification was added into the Method section, lines 119-120:

''All included lesions were >6 mm in diameter. *No other inclusion and exclusion criteria were applied, as this was a pilot study with a limited number of participants.''*

2. It is essential to specify the international classification system used to stratify samples into different groups. Did the study follow the AJCC (American Joint Committee on Cancer) melanoma staging system or the WHO classification of skin tumors? Providing this information would enhance the reproducibility and clinical relevance of the findings.

Thank you for pointing this out. Sample stratification was based on the pathologist's report which follows UICC and AJCC guidelines. This information is now added to the manuscript, lines 216-220:

'This classification of lesions is based on the pathologist's report on excised lesion samples, following the guidelines of the Union for International Cancer Control (UICC) 8th edition (2017)[47] and the American Joint Committee on Cancer (AJCC) 8th edition (2017)[48], which have been used in Sweden since January 2018[49].''

3. Did the authors observe significant differences in Kyn and Trp levels between:
Malignant melanoma vs. benign pigmented lesions (e.g., dysplastic nevi, seborrheic keratosis)?

Benign lesions vs. adjacent non-lesional skin? A stratified analysis addressing these comparisons would provide valuable insights into whether Kyn/Trp alterations are melanoma-specific or part of a broader metabolic dysregulation associated with skin lesions.

Thank you for this valuable suggestion. A comparison across different skin samples (NL, BL, MIS, and MM) was conducted using one-way ANOVA followed by a post-hoc Tukey test. A new figure 5 illustrating this comparison has been added to the manuscript (lines 269-292). The main finding is that metabolite levels gradually increase from NL to MIS and MM. However, this trend does not apply to BL (2 lesions of dysplastic nevi and 1 seborrheic keratosis lesion), where metabolite levels are even lower than in NL skin, suggesting that the increased levels of Tyr, Phe, and Trp are specific to melanoma. Interestingly, despite similar skin resistance between MM and BL (~2 times lower than in NL skin), metabolite levels were higher in MM compared to NL, whereas they were lower in BL. No significant differences were observed for Kyn levels across the samples. Further stratification and statistical comparison between NL skin and BL, including specific subtypes such as dysplastic nevi or seborrheic keratosis, was not feasible due to the small sample size. For example, only one patient was diagnosed with seborrheic keratosis, and two patients had dysplastic nevi. Future studies with a larger patient cohort are needed to conduct this type of analysis.

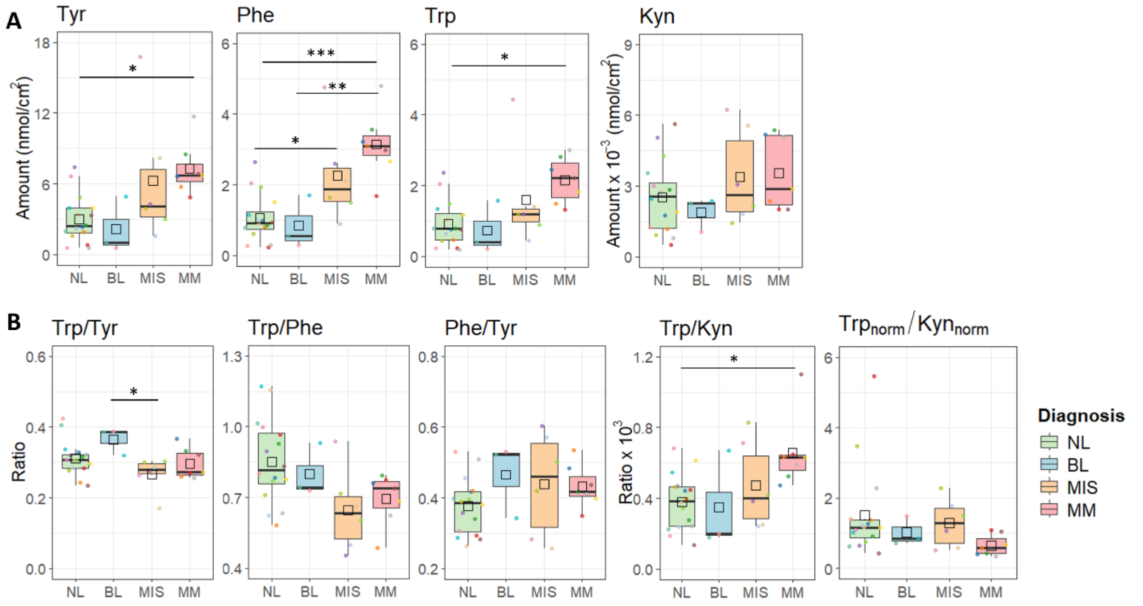

Fig 5. Comparative analysis of metabolite quantities (A) and their ratios (B) across skin surface samples (NL, BL, MIS, MM). Metabolite quantities and their ratios were compared across different skin surface samples using one-way ANOVA followed by a post-hoc Tukey test. Significance levels are indicated as: '*' $p < 0.05$, '**' $p < 0.01$, '***' $p < 0.001$.

4. How does the tape sampling technique compare to traditional methods such as punch biopsies and fine-needle aspiration? The authors should discuss the advantages and limitations of tape sampling in terms of sensitivity, specificity, and reproducibility for detecting Trp-Kyn pathway alterations.

Thank you for the question. If we understand correctly, you are asking about sensitivity, specificity, and reproducibility in detecting Trp-Kyn alterations across punch biopsies, fine-needle aspiration (FNA), and tape sampling in skin/melanoma lesions.

Currently, limited number of studies have examined Trp and Kyn levels in cancer, and we found no published data specifically measuring Kyn levels in melanoma tissue using punch biopsies, FNA, or tape

sampling (Table S9). Instead, existing studies focus on Trp-Kyn alterations in blood, Trp levels in melanoma tissue (without Kyn data), and IDO1 enzyme expression, which is linked to the Trp-Kyn pathway. Therefore, since direct comparisons between these methods to detect Trp-Kyn alterations are currently unavailable, we can only make theoretical speculations based on general features for sampling by punch biopsies, NFA and tape stripping techniques. Having said that, we have added into introduction more general comparison between punch biopsies, FNA and tape sampling for metabolite analysis, lines 71-98:

''*Several sampling methods could potentially be used to analyse Trp and Kyn in melanoma, including clinically used invasive techniques such as punch biopsy and fine needle aspiration (FNA), as well as non-invasive methods like tape stripping. However, to the best of our knowledge, no reports directly address Trp and Kyn concentrations in cutaneous melanoma tissue sampled by mentioned techniques or other methods. Punch biopsies provide full-thickness skin samples, which enable analysis of deeper tissue layers[25]. Though Trp and Kyn have not been measured in such samples, volatile metabolites [26] and lipids [27] have been successfully analysed in melanoma punch biopsies. Furthermore, studies analyzing Trp and Kyn in various models—including ex vivo diffusion studies across porcine skin[28], in vitro cell cultured skin models (measuring Trp and Kyn in both topical and basolateral compartments)[29], and investigations involving healthy human volunteers[30,31]—suggest that Kyn levels are higher in the deeper layers of the skin compared to the surface. This is important, as accurate measurement of Kyn at the skin surface is challenging. Therefore, sampling and analysis of metabolites in punch biopsies could potentially enhance reliability of Kyn quantification. Friendlier sampling FNA technique uses a thin needle to extract a small sample of tissue, which may contain cells, interstitial fluid, blood[32]. In the context of melanoma, FNA is commonly used to diagnose metastasis[33,34]. Metabolite analysis using FNA has been successfully demonstrated in thyroid cancer [35], suggesting its potential for sampling Trp and Kyn in melanoma tumors. However, FNA, as well as biopsies, is an invasive procedure and may present challenges in reproducibility due to tumor heterogeneity. In contrast, tape stripping is a non-invasive technique that avoids the risks associated with invasive sampling. It is particularly well-suited for repeated measurements and is effective for capturing surface-level metabolites, making it a promising tool for assessing Trp and Kyn levels directly on the skin surface. It is important to note that although tape stripping has not yet been applied to melanoma lesions to collect metabolites, it has been successfully used to collect genetic material for melanoma-related gene expression analysis, aiding in diagnosis [36–38]. Additionally, studies on psoriasis patients have shown that changes in metabolite profiles following treatment were detected earlier on the skin surface than in blood samples [39]. Concluding, punch biopsies and FNA might allow access to Trp and Kyn present in deeper skin layers, however tape sampling stands out as the most patient-friendly, non-invasive method and is particularly well-suited for suspected lesions screening and longitudinal studies.*''

5. Given that skin microbiota and pH can influence tryptophan metabolism, it is important to discuss whether microbial degradation of Trp into Kyn (e.g., by skin-resident bacteria such as Cutibacterium acnes) or variations in skin pH might affect the measured ratios. Were any steps taken to mitigate these confounding effects?

Thank you for this question. We agree with the reviewer that microbiota and pH could have influenced topical Trp and Kyn levels. However, microbiota composition was not assessed in the melanoma tape samples collected in this study. Since melanoma lesions assessed in this study were relatively small (~1 cm²), it is challenging to simultaneously analyze both metabolites and microbiota from the same tape sample, and further method development is required to make this approach possible. Regarding pH, our previously conducted Trp and Kyn diffusion across porcine skin upon varying pH demonstrated no significant differences in Trp and Kyn permeability across the stratum corneum (SC), except under

extreme conditions (pH 2 and pH 12). To address the potential impact of microbiota and pH on Trp and Kyn measurements on melanoma lesions, we have now included a paragraph discussing these aspects (lines 398–409):

''An additional factor that was not addressed in this study is the potential impact of skin microbiota on Trp and Kyn levels sampled from melanoma lesions. Alterations in the skin microbiota composition have been reported in melanoma[62]. Subsequently, skin microbiota may influence topical Trp and Kyn measurements in at least two ways. First, by altering the skin pH. The diffusion of permeant across SC depends on its hydrophilicity/charge, which is determined by pH driven protonation or deprotonation. Similarly, protonation/deprotonation of SC free fatty acids affects SC lipid packing and, thus, permeability through this barrier[63,64]. Second, microbiota can metabolize Trp and/or convert Trp to indoles, such as indole-3-aldehyde, as shown in other studies[65,66]. Our earlier in vitro Trp and Kyn permeability measurements, conducted over 12 hours without antibacterial agents, demonstrated substantial microbial consumption of both metabolites—at rates exceeding passive diffusion[7]. Thus, incorporating skin microbiota analysis into future studies could greatly enhance the interpretation of topical metabolic profiles in melanoma lesions.''

6.Higher kynurenine levels have also been reported in psoriasis, an inflammatory skin disease characterized by increased IDO1 expression and Trp metabolism. The discussion should include a comparison of Kyn levels in melanoma versus inflammatory dermatoses such as psoriasis, atopic dermatitis, and lichen planus. This will help differentiate whether increased Kyn is specific to melanoma or part of a broader immunological response in skin disorders.

Thank you for this comment. Trp metabolism via the Kyn pathway is indeed involved in various inflammatory skin diseases. However, as shown by Harder et al., 2016, different diseases may feature distinct enzyme expression patterns within this pathway (e.g., IDO1, KMO, KYNU) (please, see inserted figure below), potentially leading to different downstream Kyn metabolite profiles. We have now included this point in the manuscript (lines 448-451):

''The activity of Kyn pathway appears to be context-dependent. Depending on the inflammatory state or disease type, different enzymes, including IDO1, kynureninase (KYNU) metabolizing Kyn to anthranilic acid, kynurenine 3-mono-oxygenase (KMO) metabolising Kyn to 3-hydroxy-kynurenine etc., may be differentially expressed[69].''

To address this and related reviewer questions, we conducted a focused PubMed search ("tryptophan and kynurenine in skin" and "kynurenine in melanoma") and reviewed over 120 articles (S9 Table). Based on this, we expanded the discussion by including Section: ''4.2 Trp-Kyn pathway in skin and melanoma: contextualization with existing literature''. This question was addressed in the discussion, lines 438-454 and 483-495 with the main section cited below:

*''Finally, activation of Kyn pathway has been reported in other than melanoma inflammatory skin disorders, such as atopic dermatitis[83], allergic contact dermatitis[84], flaky paint (Pellagra-like) dermatosis[85], epidermolysis bullosa[86]. However, systematic Kyn levels in these conditions tend to show only mild increases, typically well below two-fold change. By comparison, our study observed an average three-fold increase in Kyn on the surface of MM lesions. Only a few studies report direct measurements of Trp-Kyn metabolites in/on defected skin (**S9 Table**). For example decreased Trp and elevated Kyn levels have been reported in hidradenitis suppurativa[87], while no differences were found between atopic dermatitis and healthy skin[66]. The lack of comparative data (**S9 Table**), and small sample size in our pilot melanoma study, limits our ability to differentiate melanoma-specific Trp-Kyn signatures from those related to non-malignant inflammation. Nevertheless, our non-invasive Trp-Kyn sampling and analysis represents a first step in assessing metabolite levels on the surface of cutaneous*

melanoma, though comprehensive studies are still needed to map and compare Trp-Kyn metabolism across skin layers and disease types.''

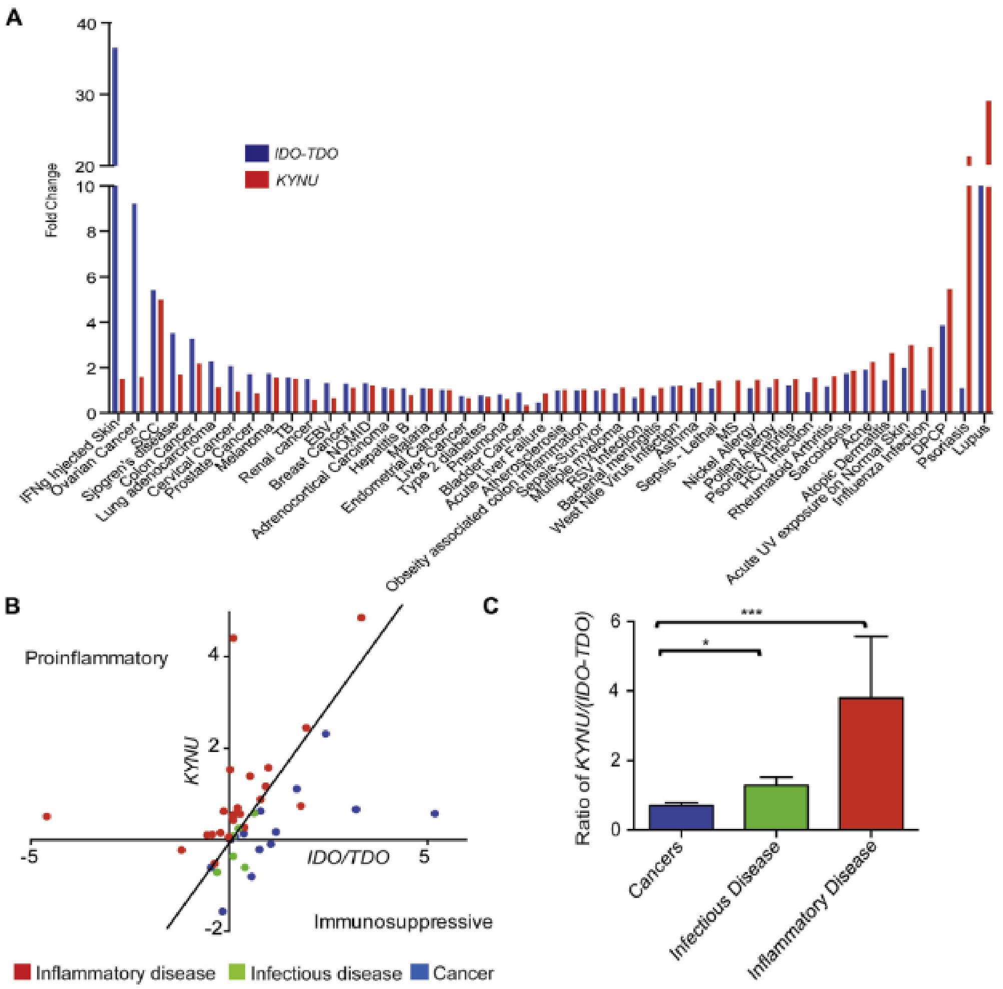

FIG 6. Human cancers and inflammatory diseases are characterized by distinct ratios of tryptophan metabolism enzymes. **A,** Publicly available gene array data from more than 40 human cancers, infectious diseases, and inflammatory diseases were mined for expression of *IDO, TDO,* and *KYNU.* The average fold change of *IDO* and *TDO (blue)* and *KYNU (red)* expression compared with that in normal or nondiseased tissue for each disease is provided. **B,** Average log fold-change of *IDO-TDO* versus *KYNU* expression for inflammatory diseases *(red)*, infectious disease *(green)*, and cancers *(blue)*. The *black line* represents a 1:1 fold-change ratio of *IDO-TDO* versus *KYNU* expression. **C,** *KYNU/IDO-TDO* ratio was pooled for cancers, infectious diseases, and inflammatory diseases (n = 9-16 samples per group). **P* < .05 and ****P* < .001.

Figure taken from Harden et al., 2016. (Harden JL, Lewis SM, Lish SR, Suárez-Fariñas M, Gareau D, Lentini T, et al. The tryptophan metabolism enzyme L-kynureninase is a novel inflammatory factor in psoriasis and other inflammatory diseases. J Allergy Clin Immunol. 2016;137: 1830–1840. doi:10.1016/j.jaci.2015.09.055)

7. Do kynurenine levels positively correlate with melanoma progression, tumor thickness , ulceration, or metastasis? If such correlations exist, kynurenine could serve as a potential non-invasive biomarker for disease staging and prognosis. The authors should provide statistical analyses supporting or refuting such associations.

Thank you for this comment. We fully agree that these correlations would provide valuable information for interpreting our data. However, since this was a pilot study with a limited number of enrolled patients, not all requested correlation analyses were feasible. For example, among the three benign lesions in our cohort, two were diagnosed as dysplastic nevi and one as keratosis; similarly, only two

patients had lymph node metastasis (Table 1), making robust correlation analyses impossible for these subgroups. Nevertheless, we explored possible correlations where data allowed (Fig. 1). Spearman correlation between Breslow thickness and the abundance of specific analytes (Fig. 1A) showed a trend of higher Phe, Trp, and Tyr levels with increasing Breslow thickness, whereas Kyn exhibited a negative correlation. However, none of these correlations reached statistical significance. When melanoma progression stage was correlated with analyte abundance (Fig. 1B), a trend toward higher Trp, Phe, and Tyr levels with more advanced melanoma stages was observed, while Kyn levels showed no clear pattern. Given the small sample size, even for these performed correlations (Fig. 1), we believe including them in the main manuscript could lead to unnecessary speculation and potentially misleading conclusions. Therefore, we chose not to include these analyses into the manuscript. We agree that further studies with larger patient cohorts will be essential to properly investigate these correlations, and this will be the next step for expanding melanoma skin surface metabolomics research. Importantly, this study has demonstrated the feasibility of metabolite sampling and analysis based on tape stripping.

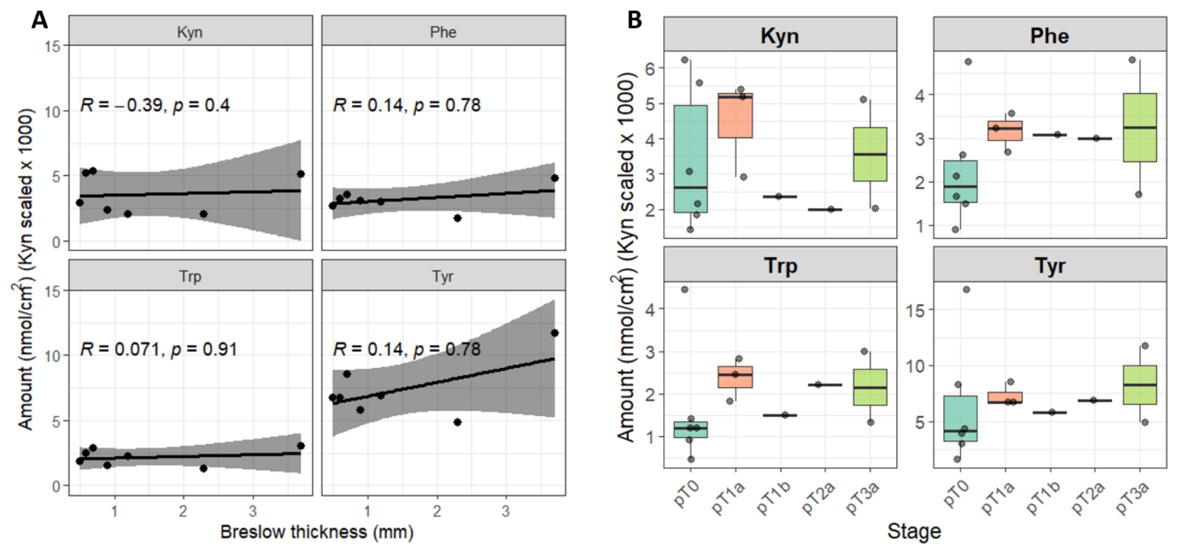

Fig. 1. Exploratory correlations between melanoma progression parameters and surface metabolite abundances. (A) Spearman correlations between Breslow thickness and analyte abundance. (B) Metabolite abundance across different melanoma stages.

8. Several pigmented skin lesions clinically mimic melanoma, including:

- Pigmented Basal Cell Carcinoma (BCC)
- Pigmented Squamous Cell Carcinoma (SCC)
- Atypical/Dysplastic Nevi
- Seborrheic Keratosis
- Blue Nevus
- Lentigo Maligna (a melanoma in situ subtype)

It would be valuable to discuss whether these lesions also exhibit increased kynurenine levels. If available, the authors should compare their findings with existing literature on Trp-Kyn metabolism in these lesions.

Thank you for this comment. We agree that this would be a very interesting comparison. However, based on our performed literature overview (S9 Table) we did not manage to identify scientific publications reporting Trp and Kyn quantification in skin tissue for the in the above-mentioned skin disorders. The overview of what we observed from the literature search are summarized in discussion section *''4.2 Trp-Kyn pathway in skin and melanoma: contextualization with existing literature''*, lines 437-495.

9.The study would benefit from a broader contextualization within existing literature. Prior studies using metabolomics approaches have examined Trp-Kyn alterations in melanoma. The authors should integrate findings from studies indexed in PubMed that have investigated Kynurenine as a biomarker for melanoma progression, The role of IDO1/TDO2 inhibitors in melanoma therapy, The impact of immunotherapy (e.g., checkpoint inhibitors) on Trp-Kyn metabolism. A comprehensive discussion comparing the current findings with previous reports would strengthen the manuscript's scientific impact.

Thank you for this suggestion. Based on performed literature search (S9 Table), the following discussion was added to the manuscript (lines 455-465 and 475-482):

''Many studies report elevated Kyn and reduced Trp levels in blood of advanced melanoma patients[11,13,71], and at other inflammatory diseases[72]. A very few have measured these metabolites directly in tumor tissue of melanoma mouse model[15], with difficulty to find an equivalent study on human melanoma tissue (S9 Table). High number of studies done on cell culture models (S9 Table), while informative, cannot replicate the metabolic complexity of the in vivo microenvironment, where metabolite concentrations are shaped by interactions with different cells, vasculature, and immune infiltrates. Furthermore, while the Trp/Kyn ratio is often used as a surrogate for IDO1 activity, this relationship is not absolute, as IDO1 may also function through non-enzymatic signaling pathways [73,74]. The Trp and Kyn metabolism might be affected by other enzymes apart from IDO1[75]. Direct quantification of these metabolites in tissue is thus essential for accurate interpretation, which currently is greatly lacking.''

''While the prevailing view supports a central role for Trp-Kyn metabolism in immune evasion, some studies caution against overestimating its importance[81] relative to other metabolic pathways involved in tumor progression [75,82]. Our own findings contribute nuance to this picture. Although we observed elevated Kyn levels in melanoma lesions, Trp levels were even more increased if compared with adjacent healthy skin. Thus, Trp/Kyn ratio sampling from the surface of cutaneous melanoma lesions have not confirmed the decrease of the ratio on melanomas. Still, signs of increased Trp catabolism within the tumor microenvironment were apparent. Combining topical and tissue-level metabolite analysis may help clarify the spatial dynamics of Trp-Kyn metabolism in melanoma.''

10. Given the role of kynurenine in immune suppression via the Aryl Hydrocarbon Receptor (AhR) pathway, could non-invasive kynurenine measurements help in monitoring response to immunotherapy? If kynurenine is a driver of immune evasion, IDO1/TDO2 inhibitors could be potential therapeutic targets. The authors should explore the clinical implications of their findings in melanoma treatment.

Thank you for an interesting comment. Based on performed literature search (S9 Table), the following discussion was added to the manuscript (lines 466-474):

''Therapeutically, the Trp-Kyn pathway has garnered interest as a target in melanoma, particularly in combination with immune checkpoint inhibitors such as programmed cell death protein 1 (PD-1) inhibitors. However, the key clinical trial on melanoma patients in Phase III using epacadostat (an IDO1

inhibitor) in combination with pembrolizumab (PD1 inhibitor) drugs, did not improve overall survival compared to placebo plus pembrolizumab[76]. Following these results, clinical trials using IDO1 inhibitors in other cancer types were also discontinued [77,78]. This setback has led to renewed efforts to understand cancer immune resistance mechanisms and to explore alternative strategies, such as enhancing Kyn degradation through enzymes like kynureninase[15,79] or blockage of AhR receptor [80], which have shown promising preclinical results in reducing tumor burden and prolonging survival.''

Overall, the study provides valuable insights into non-invasive metabolic profiling of melanoma. However, addressing the above concerns will improve the rigor, clinical applicability, and scientific impact of the findings. Providing additional data on stratified comparisons, controlling for confounding factors, and expanding the discussion on kynurenine's role in melanoma progression and immune evasion would significantly enhance the manuscript.

____

7. PLOS authors have the option to publish the peer review history of their article (what does this mean?). If published, this will include your full peer review and any attached files.

If you choose "no", your identity will remain anonymous but your review may still be made public.

Do you want your identity to be public for this peer review? For information about this choice, including consent withdrawal, please see our Privacy Policy.

Reviewer #1: No

Reviewer #3: No
