# Supplementary material for: Non-invasive tape sampling of tryptophan and kynurenine in relation to phenylalanine and tyrosine from melanoma and adjacent non-lesional skin: A pilot study
Source: PLoS One. 2025 Jun 24;20(6):e0326457. doi: 10.1371/journal.pone.0326457 (PMC12186910; doi:10.1371/journal.pone.0326457)
Supplement: Attachment — Submitted filename: Response to Reviewers.docx [file pone.0326457.s019.docx]

Contents

[**Journal Requirements**	1](#_Toc190793861)

[**Review Comments to the Author**	3](#_Toc190793862)

**Journal Requirements**

When submitting your revision, we need you to address these additional requirements.

1. Please ensure that your manuscript meets PLOS ONE's style requirements, including those for file naming. The PLOS ONE style templates can be found at

https://journals.plos.org/plosone/s/file?id=wjVg/PLOSOne_formatting_sample_main_body.pdf and

<https://journals.plos.org/plosone/s/file?id=ba62/PLOSOne_formatting_sample_title_authors_affiliations.pdf>

Followed.

2. Thank you for stating the following financial disclosure:

“The Knowledge Foundation (KK-stiftelsen), grant number 20190010; Stig and Ragna Gorthon foundation; EU Interreg ÖKS; Hudfonden (Welander Finsen foundation), the Regional research grant of the Southern health care region in Sweden; grant from the Swedish state under the agreement between the Swedish government and the county councils (the ALF agreement); the Inga and John Hain Foundation for medical research; the Eva and Ingemar Nilsson foundation; the Gyllenstiernska Krapperup Foundation; and the Gustaf Th Olsson foundation.”

Please state what role the funders took in the study. If the funders had no role, please state: "The funders had no role in study design, data collection and analysis, decision to publish, or preparation of the manuscript."

If this statement is not correct you must amend it as needed.

Please include this amended Role of Funder statement in your cover letter; we will change the online submission form on your behalf.

The funders had no role in study design, data collection and analysis, decision to publish, or preparation of the manuscript.

3. PLOS requires an ORCID iD for the corresponding author in Editorial Manager on papers submitted after December 6th, 2016. Please ensure that you have an ORCID iD and that it is validated in Editorial Manager. To do this, go to ‘Update my Information’ (in the upper left-hand corner of the main menu), and click on the Fetch/Validate link next to the ORCID field. This will take you to the ORCID site and allow you to create a new iD or authenticate a pre-existing iD in Editorial Manager.

Orcid iD was validated.

4. We note that Figure 1 in your submission contain copyrighted images. All PLOS content is published under the Creative Commons Attribution License (CC BY 4.0), which means that the manuscript, images, and Supporting Information files will be freely available online, and any third party is permitted to access, download, copy, distribute, and use these materials in any way, even commercially, with proper attribution. For more information, see our copyright guidelines: <http://journals.plos.org/plosone/s/licenses-and-copyright>.

We require you to either (1) present written permission from the copyright holder to publish these figures specifically under the CC BY 4.0 license, or (2) remove the figures from your submission:

a. You may seek permission from the original copyright holder of Figure 1 to publish the content specifically under the CC BY 4.0 license.

We recommend that you contact the original copyright holder with the Content Permission Form (http://journals.plos.org/plosone/s/file?id=7c09/content-permission-form.pdf) and the following text:

“I request permission for the open-access journal PLOS ONE to publish XXX under the Creative Commons Attribution License (CCAL) CC BY 4.0 (http://creativecommons.org/licenses/by/4.0/). Please be aware that this license allows unrestricted use and distribution, even commercially, by third parties. Please reply and provide explicit written permission to publish XXX under a CC BY license and complete the attached form.”

Please upload the completed Content Permission Form or other proof of granted permissions as an "Other" file with your submission.

In the figure caption of the copyrighted figure, please include the following text: “Reprinted from [ref] under a CC BY license, with permission from [name of publisher], original copyright [original copyright year].”

b. If you are unable to obtain permission from the original copyright holder to publish these figures under the CC BY 4.0 license or if the copyright holder’s requirements are incompatible with the CC BY 4.0 license, please either i) remove the figure or ii) supply a replacement figure that complies with the CC BY 4.0 license. Please check copyright information on all replacement figures and update the figure caption with source information. If applicable, please specify in the figure caption text when a figure is similar but not identical to the original image and is therefore for illustrative purposes only.

License was obtained from Biorender and uploaded with re-submission files.

5. Please include captions for your Supporting Information files at the end of your manuscript, and update any in-text citations to match accordingly. Please see our Supporting Information guidelines for more information: http://journals.plos.org/plosone/s/supporting-information.

Captions for supporting information were included. Track changed in revised SI version.

6. Please review your reference list to ensure that it is complete and correct. If you have cited papers that have been retracted, please include the rationale for doing so in the manuscript text, or remove these references and replace them with relevant current references. Any changes to the reference list should be mentioned in the rebuttal letter that accompanies your revised manuscript. If you need to cite a retracted article, indicate the article’s retracted status in the References list and also include a citation and full reference for the retraction notice.

Checked.

# **Review Comments to the Author**

Please use the space provided to explain your answers to the questions above. You may also include additional comments for the author, including concerns about dual publication, research ethics, or publication ethics. (Please upload your review as an attachment if it exceeds 20,000 characters)

**Reviewer #1**: The paper describes an interesting approach to answering a very common question: Is this skin "defect" a melanoma or not ? And it would be very relevant if the described tape analysis could make it easier for patients to have a correct diagnosis. Furthermore - the tape test could potentially be offered as a test you perform at home and just send the tape samples to a laboratory for analysis.

However I have quite a few comments, particularly to the standardization of the analytical method.

1. Introduction

In the introduction (Line 37-38) it is stated that over a million cases of skin cancer is diagnosed each year worldwide. The reference for this statement is ref 1, which is from 2013. But ref. 1 refers back to a paper from 2010 that summarizes numbers from 2006. Is is possible to find more recent information about the number diagnosed per year?

Thank you for this comment. The information about diagnosed skin cancer cases per year was updated by using a recently published paper (Ferlay et al., 2024) and statistics from World Health Organization (WHO) website published in 2017. Please, see lines 37-38.

Ref. 3 - please check - it must be the wrong paper in the ref list. Ref 4 - is that correct ? Please check all references carefully

Thank you. It was updated to references focusing on primarily on melanoma aggressiveness, metastasis and early detection issue. Please, see newly added references number 4 and 5. Considering reviewer comment about the refences, all references have been checked, and updated.

2.3 Metabolite extraction

In section 2.3 it is described that the three tape samples are extracted for one hour with 1 mL 20% methanol. Then 200 µL from each extraction is pooled before addition of the internal standard. I know it is too late to change this but I would definitely have added the internal standards to the 1 mL extraction solvent to also compensate for losses during extraction.

This point is well taken. We agree that adding the internal standard to the extraction solvent would have been better from a quantitative point of view. We have therefore now modified method section 2.4 to emphasize that data are presenting relative, rather than truly quantitative values. Please, see line 123.

2.4 LC-MS/MS/ analysis

In general I find it very difficult to deduct how the quantification was performed. Generally in LC-MS/MS analyses calibrators, internal controls and samples are prepared in the same way, i.e. you pipette a certain amount of sample and add a certain amount of internal standard to everything. Then the calibration curve is obtained (automatically in the LC-MS/MS system) by plotting the concentration on the X-axis and the response factor (peak area of analyte divided by peak area of internal standard).

In this paper internal standard (unknown amount of 0,25 µM) is added to combined extracts (se comment above too) of tape samples whereas the calibrators are prepared with the internal standard already added. The paper does not mention if calibrators are prepared and aliquoted to be used for different sample sets or if the calibrators are prepared every time samples are run. Or maybe the whole experiment was one single run ? Anyway if samples and calibrators were not prepared on the same day with the same solution of internal standard added in the same amount it could result in erroneous results.

Thank you, reviewer, for these comments. Clarification on sample and calibrators preparation in sections 2.3 Metabolite extraction and 2.4 LC-MS/MS analysis were added. Please, see lines 109-111, 113-117, 141-145.

Briefly, IS were added to reach 0.25 uM concentration in the samples and calibration standards for Phe, Trp and Kyn, and 0.75 uM for Tyr. We used 5uM (Phe, Trp, Kyn) and 15 uM Tyr mixture of IS. Exact volume added to the samples and calibration standards was dependent on the total volumes of the samples. Samples and calibration standards were prepared on the same day using same IS aliquot, and the experiment was done in one single LC-MS/MS run in triplicates.

Furthermore - as I read the paper - no internal controls are used. I appreciate that internal controls cannot be prepared in the same way as the samples since you cannot have multiple skin collection sites with known amount of the amino acids. But it would be interesting to see if you could extract a known amount of amino acids that were pipetted directly on the tape (in a small amount of liquid) and then extracted in the same way as the patient samples.

Thanks for this comment. Recovery and matrix effects were examined in a previous study which we now refer to in the method section (lines 109-110). It was noted in the manuscript, that different skin samples, non-lesional skin and melanoma-suspected lesions, were assumed to provide similar and high recoveries. While this can not be assessed in this study, we will refer to the following analysis as relative rather than absolutely quantitative (see lines 119-121):

In Table S2 - please adjust the number of digits after the comma to more reasonable numbers.

Thank you for the comment. In S2 Table, the values are rounded to show more reasonable numbers.

The precision of the method seems to be a measure of repeatability rather than intermediary precision since it is a measurement of the same calibration solutions three times, as I read it. The precisions obtained are therefore very low and not comparable with intermediary precisions found in the normal way where you have internal controls that are measured together with the calibrators and samples over at least 5 different days from five different sample preparation sets. The limit of quantification would normally be determined as a precision profile showing the lowest concentration that can be measured on internal controls near the LOQ with an intermediary precision below 20% in at least 5 sample sets. Here you do not have any internal controls - hence the calculation from the standard curve instead which may be acceptable.

Thank you for the reviewer’s comment. We agree that in S2 Table, we estimated repeatability rather than precision, which is now clearly stated in S2 Table and main manuscript 2.4. LC-MS/MS analysis section (please, see line 144-145).

As regards the table showing the LOD and the LOQ - please explain the two numbers before and after the slashes ?

Thank you for the reviewer’s observations. The LOD and LOQ values following the slashes represent results from a different method, which were mistakenly left in the table after that part of the study was removed from the paper. The values have now been removed.

Having said all this the exact concentration may not be very important since extract from the lesions are compared with extracts from the same patient in a healthy area. But you need to describe in more detail, so that we know you are not seeing a systematic difference between lesion type just because the samples were from individual runs with potentially different concentrations of the isotopically labelled internal standards (due to lack of stability). I hope my point makes sense …

Thank you for the comments. As indicated in our response above, we have added more details on the quantitative method used in this study. Only a partial validation of the method was conducted as the aim of the study was to distinguish between non-lesional skin/melanoma lesion rather than to provide highly accurate concentrations for amino acids on skin. Hence, repeatability of the LC-MS/MS analysis was evaluated by running calibration curve standards and samples containing spiked IS in triplicates, as well as all samples and standards were prepared the same day and measured in one LC-MS/MS run.

Results

In Table 1 there is a column called Sampling size which shows the diameter defined by adhesive frame. I am not sure how to understand this since earlier in the paper it is stated that the tape is 20 mm in diameter. So why is the sampling size less than 20 mm ?

Thank you, reviewer, for this question. As stated in the 2.1 Materials section diameter of commercially available tapes is 22 mm. However, this does not correspond to the size of melanoma suspected lesions, which varied between the patients, and usually were smaller than 22 mm in diameter. We used a specific sampling frame that matched the size of the melanoma-suspected lesions, as much as possible. A custom-made adhesive frame was employed to define sampling area and minimize the inclusion of non-lesional skin around the melanoma-suspected lesion. This is now clarified in 2.2 Experimental design section (please, see lines 92-94) and Fig. 1. caption (please, see lines 100-103).

Figures

Since the LC-MS/MS analysis has not been published previously I think you should add a Figure showing a chromatogram for for instance a calibrator and a patient sample with low concentrations of the analytes.

Thank you for this suggestion. A new figure, showing example of LC-MS/MS chromatograms obtained from standard solution and patient sample was included in Fig 3. Please, see lines 204-205.

Reviewer #2: The study is designed and executed in methodologically and medically sound manner. Clustering of malignant and benign tissues in multivariate analysis is impressive. Discussion on biological reasons of different amino acid signatures is also sound. In conclusion I would recommend this pilot study for publication.

________________________________________

6. PLOS authors have the option to publish the peer review history of their article (what does this mean?). If published, this will include your full peer review and any attached files.

If you choose “no”, your identity will remain anonymous but your review may still be made public.

Do you want your identity to be public for this peer review? For information about this choice, including consent withdrawal, please see our Privacy Policy.

Reviewer #1: No

Reviewer #2: Yes: Benjamin Benzon

________________________________________
